# Electrical Signaling of Plants under Abiotic Stressors: Transmission of Stimulus-Specific Information

**DOI:** 10.3390/ijms221910715

**Published:** 2021-10-03

**Authors:** Maxim Mudrilov, Maria Ladeynova, Marina Grinberg, Irina Balalaeva, Vladimir Vodeneev

**Affiliations:** Department of Biophysics, National Research Lobachevsky State University of Nizhny Novgorod, 23 Gagarin Avenue, 603950 Nizhny Novgorod, Russia; mtengri@yandex.ru (M.M.); ladeynova.m@yandex.ru (M.L.); mag1355@yandex.ru (M.G.); irin-b@mail.ru (I.B.)

**Keywords:** plants, electrical signals, information, reception, stimulus-specific response

## Abstract

Plants have developed complex systems of perception and signaling to adapt to changing environmental conditions. Electrical signaling is one of the most promising candidates for the regulatory mechanisms of the systemic functional response under the local action of various stimuli. Long-distance electrical signals of plants, such as action potential (AP), variation potential (VP), and systemic potential (SP), show specificities to types of inducing stimuli. The systemic response induced by a long-distance electrical signal, representing a change in the activity of a complex of molecular-physiological processes, includes a nonspecific component and a stimulus-specific component. This review discusses possible mechanisms for transmitting information about the nature of the stimulus and the formation of a specific systemic response with the participation of electrical signals induced by various abiotic factors.

## 1. Introduction

Plants cannot avoid the influence of unfavorable environmental factors due to their sedentary nature, which required their development of complex mechanisms of response to stressors. Since, in natural conditions, there is significant spatial heterogeneity in the actions of various natural factors, signaling systems play an important role in the formation of plant adaptations. Locally generated distant signals propagate throughout the plant, playing a crucial role in the formation of a coordinated response, embracing all parts of the plant’s organism. Systemic reactions including changes at the physiological, biochemical and genetic levels, and leading to the increased adaptability of plants to forthcoming stresses are covered by the term “systemic acquired acclimation” [1,2,3,4,5]. Plants have several distant signaling pathways, including hydraulic (pressure step, mass flow), electrical (Box 1, action potential [AP], variation potential [VP], system potential [SP]) and chemical (hormones, reactive oxygen species [ROS], small signaling molecules) [1,3,6]. The listed types of distant signals differ not only in their nature, but also in their propagation speed, which is low for chemical signals and high for the signals of a physical nature (hydraulic and electrical) [3,6]. This is one of the main reasons for considering chemical signaling as the main mechanism for transmitting information over short distances (to an individual cell and neighboring cells), and electrical and hydraulic signals for remote ones [7].

The propagation of distant electrical signals in plants occurs due to the actions of different stressors, including changes in temperature or light irradiation, mechanical stimuli, attack by pathogens, etc. [4,6,8,9,10,11]. It can already be confidently said that some of the changes induced by distant signal are nonspecific, i.e., a signal induced by a stressor of one nature can increase resistance to the action of another stressor. This phenomenon is referred to as cross-adaptation [12,13]. At the same time, the question arises of whether systemic responses can have specific features along with nonspecific ones. A positive answer to this question is possible only if the distant signal(s) carries information about the nature and/or intensity of the acting stimulus. To the date, several reports have been published that show the role of electrical signals in the induction of stimulus-specific responses in non-stimulated parts of the plant and make assumptions about the mechanisms underlying such specificity. However, we are far from a complete understanding of how information about the nature of a locally acting stimulus in a plant is transferred. There are several main stages in the transmission of information with the participation of electrical signals:

- the generation of a local electrical reaction (ER) with stimulus-specific features;
- the presence of stimulus-specific parameters in propagating electrical signals (ES);
- the execution of the specific functional response, depending on the parameters of a distant signal or a stimulus-specific combination of signals.

Questions regarding the nature of these stages will be analyzed in our review in relation to the distant electrical signals induced by various abiotic stressors.

### Box 1

The action potential (AP) is a systemically propagating transient depolarization with a characteristic impulse form; they possess amplitudes from several tens to one hundred mV and durations from several seconds, in locomotive plants, to several tens of seconds in ordinary plants. APs arise according to the threshold principle, obey the “all-or-nothing” law and exhibit a refractory period [1,6,9,10,11]. The generation of an AP is associated with the activation of voltage-dependent Ca^2+^ channels, the molecular nature of which remains uncertain [14].

Calcium causes the activation of anion channels, simultaneously with the deactivation of the H^+^-ATPase of the plasmalemma, which leads to the formation of the AP depolarization phase. The release of K^+^ and reactivation of the H^+^-pump form the repolarization phase [1,6,9,10,11]. An AP, being a self-propagating electrical signal, is transmitted over long distances at a speed of 1–10 cm/s through phloem elements and to neighboring cells through membrane bonds in plasmodesmata [1,6,8,9,10,11,15]. The propagation of the electrical impulse causes shifts in the concentrations of AP-forming ions in the cells where it was generated. Such ions, in particular, Ca^2+^ and H^+^, are the most important regulators of intracellular processes responsible for the induction of AP-related systemic responses [1,6,8,10].

Variation potential (VP), otherwise termed as “slow wave potential” [8,16], is a transient depolarization of an irregular shape, with an amplitude of several tens of mV and a duration of up to several tens of minutes [1,6,8,9,10]. Unlike an AP, a VP does not obey the “all-or-nothing” law and differs in amplitude and the duration of depolarization, depending on the generating stimulus. Also, a VP is not a self-propagating electrical signal, but is a local electrical response induced by the propagation of a hydraulic wave and/or a chemical agent, i.e., a combination of hydraulic and chemical signals is the probable mechanism of VPs’ propagation [1,6,8,10,11,17]. ROS can act as chemical agents, the self-propagating waves of which are associated with VP propagation [1,6,8,11]. The initiation of a VP is associated with the activation of ligand-gated or mechanosensitive calcium channels. In particular, the role of GLR3.1, 3.2, 3.3, and 3.6 in the formation of a VP has been suggested [18,19]. The leading role in the formation of the depolarization wave is played by the calcium-induced decrease in the activity of H^+^-ATPase, and the duration of the shifts in ionic concentrations, in the case of VPs, is much longer than those of APs [1,6,8,9,10,11,17].

A system potential (SP) is a systemically propagating change in membrane potential towards hyperpolarization, of various amplitudes and durations. The mechanism of SP development is presumably the activation of H^+^-ATPase. This type of signal is the least studied and its induction is observed under very specialized conditions [6,20].

## 2. Stimulus Perception

### 2.1. Temperature

Temperature is one of the most important environmental factors determining the growth and development of plants. Its changes, in natural conditions, can be quite significant, and the adaptation of plants to temperature depends on intracellular and extracellular signaling, including electrical signals.

#### 2.1.1. Cold

**Figure 1:**
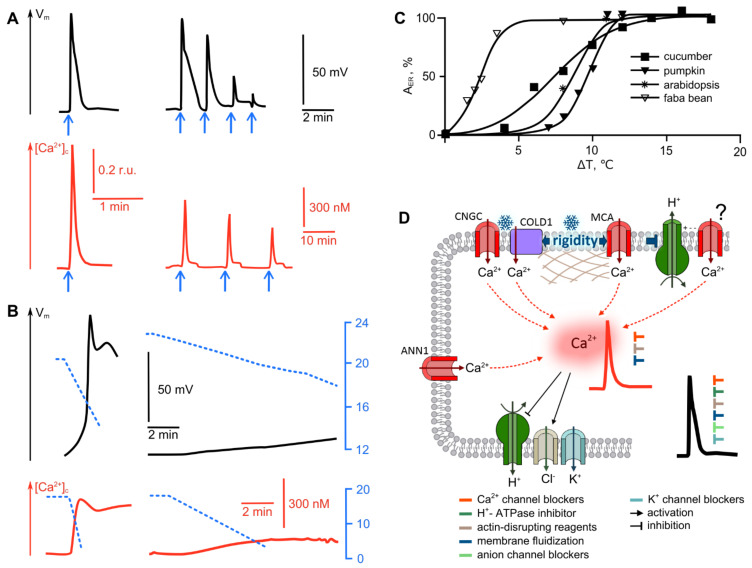
Cold-induced electrical reaction (ER): (**A**) Impulse cooling: the left shows the change in the electric potential [29] and cytosolic concentration of free Ca^2+^ ([Ca^2+^]_c_) [30] for a single stimulus, and the right shows changes for repetitive stimuli (V_m_ [29], [Ca^2+^]_c_) [31]; (**B**) gradual cooling: the left shows the change in the electric potential [15] and [Ca^2+^]_c_ [31], induced by rapid cooling, and the right shows those induced by slow cooling (V_m_ [23], [Ca^2+^]_c_) [31]; (**C**) dependence of the ER amplitude (in % of the maximum amplitude) on the depth of impulse cooling in various plant species: based on data from arabidopsis [21], faba bean [22], cucumber [23], pumpkin [25]; (**D**) hypothetical scheme of the generation of cold-induced ER. Explanations provided in the text. Black lines indicate the ER, red lines—[Ca^2+^]_c_ dynamics, blue lines—dynamics of temperature. V_m_ is the membrane potential. Blue arrows indicate the moment of cooling stimulation.

The vast majority of studies of cold-induced electrical reactions have been performed using two modes of stimulation: rapid cooling pulses and gradual cooling at a fixed rate. In the first case, the electrical response represents a transient depolarization in the form of a single impulse (Box 1, Figure 1A) [21,22,23,24,25]. The parameters of the electric impulse depend on the type of plant under study and the area of stimulation. On average, when cooled with ice water, the amplitude of an ER is about 60–70 mV, and its pulse duration does not exceed a minute for non-locomotive plants and a few seconds for locomotive ones [18,26,27,28]. Pulse depolarization in the cooling zone does not follow the “all-or-nothing” characteristic of APs, but is gradual in accordance with the amplitude (and rate) of temperature drop [21,22,23,24,25]. The dependence of the ER amplitude on the depth of cooling is displayed by a typical sigmoid curve with saturation; to reach the half-maximum ER amplitude, a temperature drop of only a few degrees is required (Figure 1C). In addition to gradualness, electrical reactions in the cooling zone demonstrate desensitization, the property characteristic of receptor potentials when repetitive stimuli induce electrical pulses of lower amplitudes (Figure 1A) [23,29]. Rapid cooling pulses induce APs, spreading beyond the local irritation zone with high probability [25,28].

Slow gradual cooling at a rate not exceeding ten degrees per minute also causes the transient depolarization of cells. The distinctive feature of the electrical reaction during slow cooling is weak depolarization; upon reaching a threshold level, an AP is generated, usually a single pulse, but in some cases a series of repetitive pulses (Figure 1B) [15,32]. In the case of slow gradual cooling, an accommodation typical for receptor potentials is well manifested: the threshold depolarization value for the appearance of an AP depends on the cooling rate. A deceleration of the cooling causes an increase in the AP-generation threshold [15], and lowering the rate below the critical level, which is about 0.5 °C/min, leads to smooth, low-amplitude depolarization without the generation of a manifested impulse [23,24,25].

The influx of calcium to cell cytosol is likely to play a pivotal role in cold-induced changes in electrical potential. This is convincingly evidenced by experimental data of inhibitory analysis using calcium channel blockers and Ca^2+^ chelators. The application of the Ca^2+^ channel blockers, including LaCl_3_ [22,23,33], neomycin [29,33,34], ruthenium red [33] and verapamil [29,35], causes significant suppression of the electrical response, as shown in a number of plants species. The exception of Ca^2+^ from the extracellular medium with the use of the chelator ethylene glycol-bis(2-aminoethylether)-N,N,N′,N′-tetraacetic acid (EGTA) leads to a similar effect [23,35]. Cold-induced depolarization is also inhibited by anion channel blockers such as ethacrynic acid [35], anthracene-9-carboxylic acid (A-9-C) [33], and 5-nitro-2-(3-phenylpropy1amino)-benzoic acid (NPPB) [36]. The formation of the repolarization phase of the cold-induced electrical reaction is likely to be contributed by K^+^-channels, as evidenced by an increase in pulse amplitude under the action of tetraethylammonium (TEA) [33,34]. Another participant in the formation of an electrical reaction is the plasmalemma proton pump, which is confirmed with H^+^-ATPase inhibitors [32,35]. Of note, both the H^+^-ATPase activity and the probability of anion channels opening are controlled by the concentration of intracellular calcium [36].

The pivotal role of Ca^2+^ in the generation of cold-induced electrical reactions is supported by obvious similarity in the simultaneously recorded dynamics of the cytosolic concentration of free Ca^2+^ ([Ca^2+^]_c_) and the membrane potential [21,36]. In the case of rapid cooling, the calcium signature has a characteristic pulse shape, with a duration not exceeding several tens of seconds (Figure 1A,B) [21,36,37,38]. Similarly, for the electrical response, the amplitude of the calcium peak depends on the depth of cooling [21,31]. Desensitization also occurs, manifesting as a decrease in the amplitude of the calcium peak with repeated cold shock [31] (Figure 1A). When gradual cooling is applied, there are single or repeated spikes of calcium concentration after the temperature reaches a certain threshold value, and a significant decrease in the cooling rate results in the absence of a characteristic peak of [Ca^2+^]_c_ [31], similarly to electrical reactions (Figure 1B). It should be noted that the source of calcium entry into the cytosol, apparently, can be both extra- and intracellular stores, as evidenced by the incomplete suppression of the Ca^2+^ signature during chelation of extracellular Ca^2+^ with EGTA [30], and the pronounced effect of agents, known as inhibitors of Ca^2+^-channels, located on the inner cell membranes [39,40].

Intensive molecular genetic studies, carried out in recent years, have made it possible to identify and systematize the genes of ion channels responsible for calcium conductivity in plants; the information on the diversity of calcium channels is summarized in an excellent recent review [14]. The ion channels responsible for calcium conductance are represented by several families: cyclic nucleotide-gated channels (CNGC), ionotropic glutamate receptors (GLR), two-pore channels (TPC), annexins (ANN) and mechanosensitive channels (‘Mid1-Complementing Activity’ channels [MCA], ‘mechanosensitive channels of small [MscS] conductance-like channels (MSL) and hyperosmolality induced [Ca^2+^]_c_ channel [OSCA]). Studies performed on mutants deficient in a certain type (or combination) of calcium channels have made a significant contribution to the deciphering of the mechanisms of stimuli perception. Thus, mutant *Arabidopsis* plants, deficient in the mechanosensitive calcium channels MCA1/MCA2, demonstrated a decrease in cold-induced Ca^2+^ influx, along with a reduction in cold resistance [30]. CNGC14 and CNGC16, from the family of cyclic-nucleotide-gated channels, were proven to participate in the formation of calcium spikes upon cooling in rice plants [41]. The role of these channels is also confirmed by the suppression of Ca^2+^ entry, in the presence of an inhibitor, ruthenium red, considered a selective blocker of cyclic ADP-ribose-dependent Ca^2+^ influx [33], as well as by an increase in their expression under cold exposure [42,43]. A decrease in cold-induced Ca^2+^ influx has recently been shown in annexin 1 (ANN1)-deficient Arabidopsis plants [44]. However, a lower degree of calcium spike inhibition should be noted for ANN-deficient mutants, as compared to MCA and CNGC mutants [30,41,44]. Another potential player in cold response-induction is the COLD1 protein [45], found in rice (*Oryza*) and proposed as one of the primary cold sensors, discussed below. COLD1-deficient mutant plants show significant suppression of the cold-induced Ca^2+^ signature, while their response to NaCl or water at room temperature is unaffected [45].

Taken together, these data may indicate the complex nature of the cold-induced calcium signal, the formation of which may be due to the activation of several types of channels. This assumption is confirmed by an additional reduction in Ca^2+^ influx in mutant plants treated with Ca^2+^-channel inhibitors. The latter was reported for MCA1/MCA2 mutants, with the addition of La^3+^ [30], and forANN1 mutants treated with La^3+^ and, especially, Gd^3+^, a well-known blocker of mechanosensitive channels [44].

The totality of the obtained data convincingly indicates the activation of a number of Ca^2+^ channels upon cooling; however, the question arises as to what causes their activation, i.e., what is the primary cold sensor. The currently accepted model assumes that primary reactions include changes in membrane fluidity and the rearrangement of the cytoskeleton [42], followed by an influx of calcium, which triggers downstream events. Cold-induced changes in the viscosity of the lipid bilayer of membranes or individual lipid rafts are considered mechanisms of activation of mechanosensitive MCA1/MCA2 [37,42,46,47]. This is supported by the suppression of Ca^2+^ influx after the fluidization of membranes with benzyl alcohol under cold stress conditions [47], along with the suppression of cold-induced gene expression [40]. The opposite effect was observed when increasing the rigidity of the membrane using dimethyl sulfoxide (DMSO) [40].

Another protein sensitive to changes in membrane rigidity or directly perceiving cooling, at least in rice plants, is COLD1 [42,45,48]. COLD1, which is a transmembrane protein, interacts with the a-subunit 1 of the G-protein (RGA1), leading to an increase in GTPase activity and the influx of Ca^2+^ to the cell [42,45].

Decreasing the activity of the plasmalemma H^+^-ATPase, leading to depolarization, may be another way the cold-induced increase in membrane viscosity affects electric potential. The hypothesis is confirmed by similar inflection points of the temperature dependences of the activity of H^+^-ATPase, the electrical response threshold, and conformational rearrangements of the lipid bilayer [32,49]. In this case, reaching the threshold level of depolarization causes the activation of voltage-dependent Ca^2+^ channels and the generation of an AP. The biophysical characteristics of such Ca^2+^ channels have been well established by analysis of the depolarization-activated calcium conductivity (DACC) of the plasmalemma of plant cells; however, DACC is still not associated with any gene [14].

The rearrangement of the cytoskeleton is discussed as an alternative direct temperature sensor, which is supported by the correlation of a critical temperature for microtubule rearrangement in plants’ cold sensitivity [50,51], and a good correspondence between the Ca^2+^ influx and the level of microtubule organization in cold conditions [52]. The cold-induced depolymerization of microtubules is a well-documented phenomenon in many plant species (for a review, see [51]), presumably causing the activation of mechanosensitive channels [40,51,52]. It is assumed that it is the destruction of actin microfilaments and tubulin microtubules that initiates Ca^2+^ entry into the cell [52], which puts cytoskeleton depolymerization downstream in the cold-perception pathway. Despite the need to further study the detailed sequence of events triggered in plants by cooling, there is no doubt about the need for a native cytoskeletal structure [22,47] for cold-induced changes in Ca^2+^ concentration and the induction of electrical response.

Thus, the sequence of events in the generation of a cold-induced reaction can be represented as follows (Figure 1D). At the initial step, a decrease in temperature causes changes in the rigidity of membranes or lipid rafts and rearrangement of the cytoskeleton. Such changes can lead to the activation of the mechanosensitive Ca^2+^ channels, MCA1/MCA2, as well as to the reduction of H^+^-ATPase activity. Additionally, the activation of the calcium channels CNGC14, CNGC16, and ANN1 appears to occur, the mechanisms of which have not been studied yet. The COLD1 sensor plays an important role in the provision or regulation of cold-induced calcium conductivity. The activation of the corresponding channels lead to the formation of a characteristic cold-triggered [Ca^2+^]_c_ signature. In turn, the dynamics of intracellular Ca^2+^ concentration controls the activity of potential-determining ion transport systems, such as H^+^-ATPase and anion and potassium channels. The activation of anion channels and inactivation of ATPase lead to forming the depolarization phase of a cold-induced electrical pulse. The repolarization phase is formed with the participation of potassium channels and, due to the reactivation of H^+^-ATPase, as a consequence of a decrease in Ca^2+^ concentration in the cytosol. Exceeding the depolarization threshold level results in the generation of an AP, which propagates beyond the local cooling zone.

#### 2.1.2. Heat

**Figure 2:**
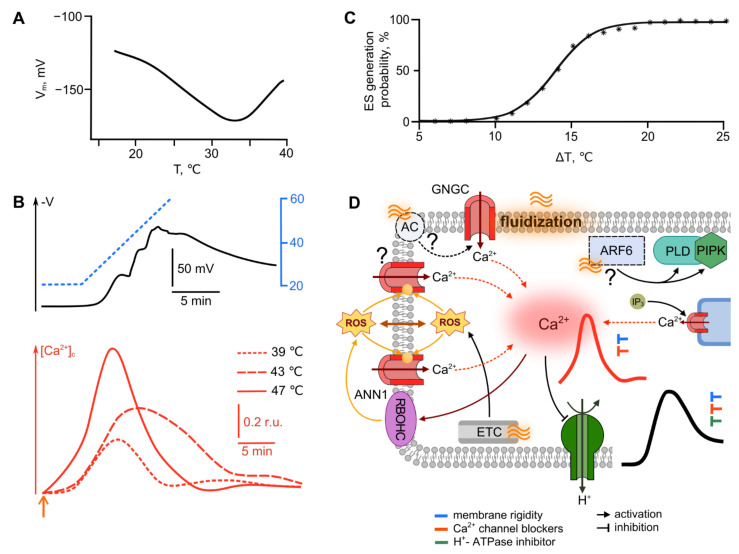
Heat-induced electrical reaction (ER): (**A**) the temperature dependence of the membrane potential of pumpkin cells [15]; (**B**) a heat-induced ER in wheat plants (unpublished data) and [Ca^2+^]c dynamics at different stimulus temperatures [57]; (**C**) the dependence of the electrical signal (ES) generation probability on the degree of heating in the Venus flytrap [58]; (**D**) a hypothetical scheme of the generation of heat-induced ERs. Explanations provided in the text. Black lines indicate the ER; red lines—[Ca^2+^]_c_ dynamics; blue lines—the dynamics of temperature. V_m_ is the membrane potential. V is the extracellular (surface) electric potential. Orange arrow indicates the moment of heating stimulation.

Local changes in the electric potential under high-temperature stress are less studied in comparison with cooling. Gradual moderate heating leads to membrane hyperpolarization, followed by depolarization when an optimum is exceeded, which, for thermophilic plants, is more than 30 °C [15,24,53] (Figure 2A). Rapid heating to high temperatures causes the formation of a deep depolarization, followed by slow repolarization (Figure 2B). The electrical reaction propagating from the local heating zone is a typical VP (Box 1) [53,54,55]. It should be noted that the temperature difference required for the induction of the ES, in the case of heating, is much greater in comparison with cooling and, apparently, amounts to several tens of degrees. The mechanisms of ER generation directly in the local heating zone have, practically, not been studied, but it is assumed that it is related to the modulation of H^+^-ATPase activity [15,49,56].

Similar to the cold, heat shock induces the entry of Ca^2+^ into the cell [57,59,60,61], which can serve as a trigger for an electrical response. The calcium wave induced by heating is characterized by a longer duration (significantly exceeding that for cooling) and an extremely slow return to initial levels [41,57,59,62,63,64] (Figure 2B). The parameters of the calcium peak demonstrate temperature dependence: the higher the temperature, the greater calcium influx, and with a smaller observed lag period [57,64]. Pharmacological analysis unambiguously shows a role of extracellular Ca^2+^ in the generation of such signals, the influx of which is inhibited by the blockers of the plasmalemma calcium channels LaCl_3_, 2-aminoethyldiphenyl borate (2-APB) and flufenamic acid (FFA), and by Ca^2+^ chelators [57,63,64,65]. The data on the role of intracellular calcium stores are more contradictory, since different scientific groups report both the effect of ruthenium red, an inhibitor of intracellular Ca^2+^ channels [57], and the absence of any effect [64].

Ca^2+^ channels from the CNGC family, including CNGC2, CNGC6 from *Arabidopsis thaliana*, and their putative ortholog CNGCb from *Physcomitrella patens*, are assumed to be the main participants in the formation of a calcium wave upon heating. For these channels, participation in the elevation of cytosolic Ca^2+^ with increasing temperature was experimentally proven [62,66,67], along with role in the heat-induced gene expression and thermotolerance. Also, at least for *Physcomitrella patens*, the presence of two more heat shock responsive calcium channel of an unknown type was shown; and the intensity of Ca^2+^ influx was higher for plants deficient in CNGCb. The latter observation can be explained, firstly, by the role of CNGCb as a non-crucial, replaceable subunit preventing over-activation of these two unidentified channels, and secondly, by the possible compensation for the loss of the CNGCb by overexpression of these unidentified channels [62]. Other candidate channels from the same family are CNGC14 and CNGC16: mutant rice plants deficient for these channels demonstrate the inhibition of heat-induced Ca^2+^ entry [41]. A number of studies also demonstrate the possibility of involvement of annexins ANN1, ANN2 and ANN4 in the regulation of the heat-induced response [63,68,69].

The primary reception of heat shock can be related to the functioning of several systems. In particular, it has been suggested that the activation of the CNGC family channels may be mediated by cyclic adenosine (cAMP) or guanosine monophosphate (cGMP) produced by an unknown adenylate cyclase or guanylate cyclase [41,66]. However, an enzyme with such an activity has not been identified. Moreover, the presence of adenylate and guanylate cyclases in plants is a subject of discussion, although we must note that in recent years a number of candidate genes have been identified (for reviews, see [70,71]). It has been demonstrated that, upon heating, phospholipase D (PLD) and phosphatidylinositol-4-phosphate-5-kinase (PIPK) are activated, followed by the accumulation of various signaling molecules, such as phosphatidic acid, phosphatidylinositol-4,5-bisphosphate (PIP_2_) and D-myo-inositol-1,4,5-triphosphate (IP_3_). Phospholipase C9 is also supposed to participate in the perception of heat; its reduced activity in mutant plants leads to both a decrease in IP3 production and a diminution in thermotolerance [72,73]. Phospholipase C can regulate the activity of Ca^2+^ channels and the response triggered by Ca^2+^ influx [57,74,75]. The G-protein ARF6 is assumed to be an upstream participant in the PLD and PIPK signaling cascades, but this assumption needs to be experimentally proven [74].

It cannot be ruled out that, as in the case of a decrease in temperature, the viscosity of the membrane acts as the primary receptor for the increase in temperature. This is indicated by the similarity of effects of temperature and benzyl alcohol, which causes the fluidization of membranes, on the concentration of intracellular calcium and the expression of chaperones, which is a typical response to heat shock [65].

When considering plants’ heat-induced response, it is impossible not to note the involvement of ROS, the levels of which are significantly increased under heating [61,76,77,78].

The generation of ROS has been shown to depend on the intensity of heating; and that NADPH oxidases’ inhibitor, diphenyleneiodonium chloride (DPI), suppresses ROS accumulation [78]. ROS produced by membrane NADPH oxidases are likely to control the functioning of annexins, possibly through the homologue of respiratory burst oxidase C (RBOHC), which makes annexins secondary heat-shock sensors [79,80]. Along with the enzymatic pathways of ROS generation under heat stress, the contribution of mitochondrial and chloroplast electron transport chains (ETC) is important [81,82]. Nitrogen oxide (NO) can also participate in the response to heat shock by cross-talk with ROS, regulating both ROS levels and the activity of downstream pathways [83]. In addition, recent studies have shown that NO production can be controlled by Ca^2+^ entering through the CNGC6 channel [84].

It is necessary to note the role of denatured proteins in the perception of heat shock, which trigger heat-responsive gene expression through the endoplasmic unfolded protein response (UPR) [85,86] and the cytosolic protein response (CPR) [87]. However, proteins responses are downstream events in the signaling cascade, in relation to Ca^2+^ and ROS [67,72,85,87]. Phytochrome B (phyB) is also considered a primary heat receptor, since the rate of phyB inactivation and reactivation in the dark is proportional to temperature. Of importance, phyB can temperature-dependently directly bind to the promoters of key target genes; however, the characteristic times of these processes are too slow, compared with rates of ER formation [42,48,88,89].

In total, the data available to date are insufficient to form a complete picture of the mechanism of heat perception and the generation of heat-induced ERs; but the following sequence of events can be assumed (Figure 2D): heat increases the fluidity of the plasma membrane and induces Ca^2+^ influx into the cytoplasm via CNGC and/or ANN. Another important event is the accumulation of ROS due to the altered functioning of enzymatic systems (for example, RBOHC) and the disruption of mitochondrial and chloroplast ETC, which can also lead to an increase in cytoplasmic calcium. The initial events result is the formation of a wave of increased [Ca^2+^]_c_, the duration of which significantly exceeds the [Ca^2+^]_c_ peak under cold stimulation. Changes in the activity of ion transport systems, which are probably under the control of intracellular calcium, cause the formation of deep, long-term depolarization. Due to the universal nature of the depolarization mechanisms in plant cells, it can be assumed that, along with H^+^-ATPase, the role of which has been proven experimentally, anion channels also may contribute to the formation of depolarization.

### 2.2. Light

Light is one of the most important factors that controls the growth and development of plants. It is not only a source of energy, but also an important regulator of biochemical and physiological processes. Distant electrical signalization is among the mechanisms for whose regulatory function light is responsible [4,90]. ES generation, in response to changes in illumination has been demonstrated long ago and for many plant species [4]. Despite this, the mechanisms of ES induction by light have not been studied enough to form a holistic picture. In this review, we summarize the known information about light-triggered ES and put forward hypotheses about its underlying mechanisms.

**Figure 3:**
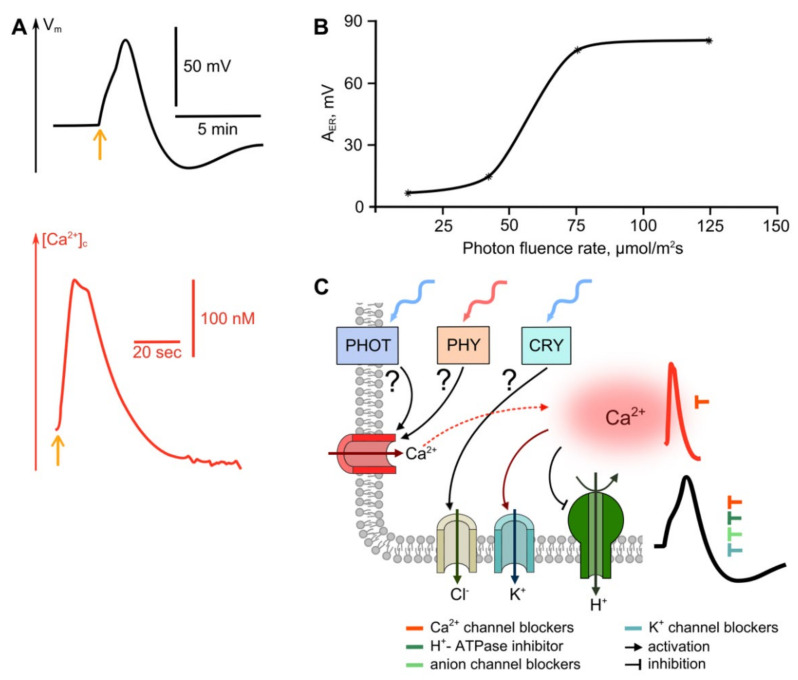
Light-induced electrical reaction (ER): (**A**) light-induced ER [96] and [Ca^2+^]_c_ dynamics [103] (Copyright (1999) National Academy of Sciences, U.S.A.); (**B**) the dependence of ER amplitude on the fluence rate of a light pulse [91]; (**C**) a hypothetical scheme of the generation of light-induced ER. Explanations provided in the text. Black lines indicate the ER, red lines—[Ca^2+^]_c_ dynamics. V_m_ is the membrane potential. Yellow arrow indicates the moment of light stimulation.

Changes in the electrical potential induced by changes in lighting conditions show significant variability depending on the intensity, spectrum and duration of illumination, as well as on the plant species and the functional cell’s specialization [15,91,92,93,94,95]. A typical reaction to light is the transition of the electric potential to a new hyperpolarized level, which is preceded by a short-term pulse depolarization [15,34,91,92,94,95,96,97,98,99] (Figure 3A). Illumination-induced depolarization has a number of features characteristic of receptor potentials. First of all, it is ER amplitude dependence on light intensity [91,92,95,100] (Figure 3B). Reaching the threshold value leads to the generation of an AP obeying the “all-or-nothing” law, capable of propagating throughout the plant with a refractory period [95,97,100,101,102]. Threshold values of light intensity for AP generation are about 80 μmol m^−2^ s^−1^, which does not exceed natural illumination conditions [94,95,100]. Similar to the reactions described above, light-induced ERs exhibit desensitization upon repeated light stimuli [100].

The mechanism of formation of light-induced ER has not been completely investigated, and the available information is contradictory. One of the possible reasons for this may be the revealed differences in the mechanisms of the generation of light-induced ER in cells with certain functional specializations [92,98]. The key stage in the formation of the reaction, apparently, is the activation of Ca^2+^ channels, which causes calcium’s entry into the cell and the subsequent depolarization thereof [92,93,98,99,101,103,104]. This is supported by the suppression of the ER by La^3+^ and nifedipine [91,94]. In addition to Ca^2+^, an alternative mechanism of light-induced depolarization is the flux of anions, in particular Cl^−^, resulting from the activation of anion channels [92,93,98]. It has been shown that inhibitors of the anion channels A-9-C and NPPB reduce the amplitude of light-induced ER in *Arabidopsis* plants and *Conocephalum* moss, although they do not completely suppress it [99,105]. However, on *Physcomitrella* moss, the effect was demonstrated for NPPB but not for A-9-C [91]. 

The role of K^+^ channels is not entirely clear. In a number of studies, the use of TEA, an inhibitor of K^+^-channels, suppressed the induction of the depolarization spike under red light illumination; however, the reaction was restored when K^+^ was replaced by Na^+^ in the medium [91,99]. On the other hand, several other reports suggest the participation of K^+^ channels in the formation of the repolarization phase and long-term hyperpolarization [34,93,103]. H^+^-ATPase is also among the participants in the formation of light-induced ER. It contributes to the formation of the pulse repolarization phase, as well as the subsequent hyperpolarization [92,93,98]. This is confirmed by the dynamics of the pH of apoplasts under the influence of blue and red light [106]. It has also been suggested that inhibition of H^+^-ATPase activity contributes to the formation of the depolarization phase of light-induced pulses [101,107].

Changes in illumination, similar to other stimuli, causes a transient increase in the concentration of intracellular calcium. The duration of the calcium peak is seconds to several minutes (Figure 3A) [93,103]. The spectral dependence of the calcium peak amplitude has been demonstrated, with two maxima at wavelengths of 440 and 470 nm [103]. As in electrical responses, Ca^2+^ peaks show desensitization [103]. At the same time, it should be noted that there is a high diversity in the [Ca^2+^]_c_ dynamics described in various works, including the presence of several calcium waves, as well as the absence of a Ca^2+^ rise [36,92,93,103]. The mentioned diversity may be due to the functional specialization of cells in which Ca^2+^ dynamics are registered, as well as to peculiar features of individual plant species.

To date, the perception of light of various spectral composition has been studied quite well, and the systems responsible for primary light perception and the activation of signaling cascades have been identified. These systems include cryptochromes, phytochromes and phototropins. Cryptochromes are responsible for the perception of blue, partly ultraviolet and green light [108,109,110], phototropins for the perception of blue light [108,110,111] and phytochromes are responsible for red and far-red light [110,112]. Along with the activation of specialized receptor systems, light-induced changes in membrane electric potentials can also be caused by the activation of photosynthetic processes. In this regard, first of all, it is necessary to consider the spectral dependence of an ER, although we must note that the experimental data are rather contradictory. There is work that has shown the absence of ER under illumination with any light except blue [107]; in other work, the induction of ER under illumination with red light was shown [91,96,99]. In the latter case, ER generation under red light was not associated with the functioning of the photosynthetic apparatus, since transient depolarization was observed in etiolated plants and when the photosystem II was inhibited using DCMU [91,92]. Finally, the results of several studies demonstrate the significant suppression of light-induced ER by 3-(3′-4′dichlorophenyl)-l,l-dimethyl urea (DCMU) [95,100,101], as well as the similarity of ER amplitudes’ spectral dependence and the spectra of photosynthetic pigments [102].

Concerning the direct involvement of specialized receptor systems, there is evidence for the role of cryptochromes, previously defined as HY4, in the generation of blue light-induced ER, which is suppressed in deficient mutants. The generation of ER is presumably caused by the activation of anion channels, which is confirmed by the effect of the NPPB anion channel blocker, similar to that of mutant plants [113]. It can be assumed that it is cryptochromes that are responsible for the specificity of the activation of anion channels, observed under blue light, and absent under red [105]. Of note, the possibility of anion channels’ activation by blue light without the participation of Ca^2+^ has been demonstrated [36]. Also, it has been proposed that the contribution of cryptochromes to the ER induction by blue light can be controlled via phototropins [114]. For the case of red light, the role of phytochromes in the induction of ERs was suggested, based on the data on ER generation under illumination with red light and its suppression by simultaneous illumination with red and far-red light. The red light-induced depolarization in this case is associated, apparently, with calcium entry to the cell [91,104]. Under the action of blue light on mutant plants, the activation of Ca^2+^-channels was shown with the participation of phototropins, but not cryptochromes [115]. One of the most important participants in light-induced electrical reactions is the H^+^-ATPase of the plasma membrane. H^+^-ATPase activity is stimulated by phototropins under blue light [116]. Presumably, phototropins can interact with H^+^-ATPase through blue light signaling 1 (BLUS1) protein kinase and an unknown component, possibly a protein kinase or phosphatase, which interacts with the phosphorylation sites of H^+^-ATPase and provides the attachment of 14-3-3 proteins that activate the H^+^-ATPase [117].

Signal induction by light is possible not only due to the activation of specialized receptor systems, but also as the result of the action of light of very high intensity, which apparently leads to cell damage. The induction, by excess light, of a propagating ROS wave generated with the participation of RBOHD has been demonstrated; it was confirmed both in mutant plants and by the suppression of ROS-wave propagation by DPI, the inhibitor of RBOH [94,118,119]. Of importance, the propagating ROS wave probably underlies the generation of such a typical electrical signal for damaging stimulation as VP (Box 1). H_2_O_2_ production under light stress is associated with cells located along the vessels; ROS production demonstrates two phases, and it is assumed that three regions of the photosynthetic apparatus are implemented in ROS generation: the light-harvesting complex of photosystem II (PSII), the reaction center of PSII, and the acceptor site of photosystem I (PSI). ROS production depends on the degree of damage to the photosynthetic apparatus [94,120,121,122] resulting from light-caused heating.

Thus, the sequence of events during the induction of ES by light can be represented as follows (Figure 3C): light leads to the activation of calcium and/or anion channels, the coding genes of which have not been identified. Under blue light, the activation of anionic channels is apparently associated with cryptochromes, and calcium channels with phototropins. Under red light, the activation of calcium channels is possible, associated with phytochromes. The functioning of anionic and/or calcium channels cause the membrane depolarization. The repolarization phase of the pulse, as well as the subsequent prolonged hyperpolarization, is probably a result of the activation of proton ATPase and potassium channels.

### 2.3. Perception of Mechanical Stimuli

One of the most common factors acting on plants in natural conditions is mechanical stimulation. Mechanically induced stresses are experienced by both the shoot and the root, due to gravity, wind, animal activity, soil hardness, etc. Local mechanical stimuli require rapid responses from the plant, both in the zone of stimulation and beyond. As with most other stimuli, regardless of modality, mechanoreception is characterized by the absence of specialized sense organs. The only exceptions are, apparently, specialized receptor hairs of the traps of insectivorous plants [123,124,125].

**Figure 4:**
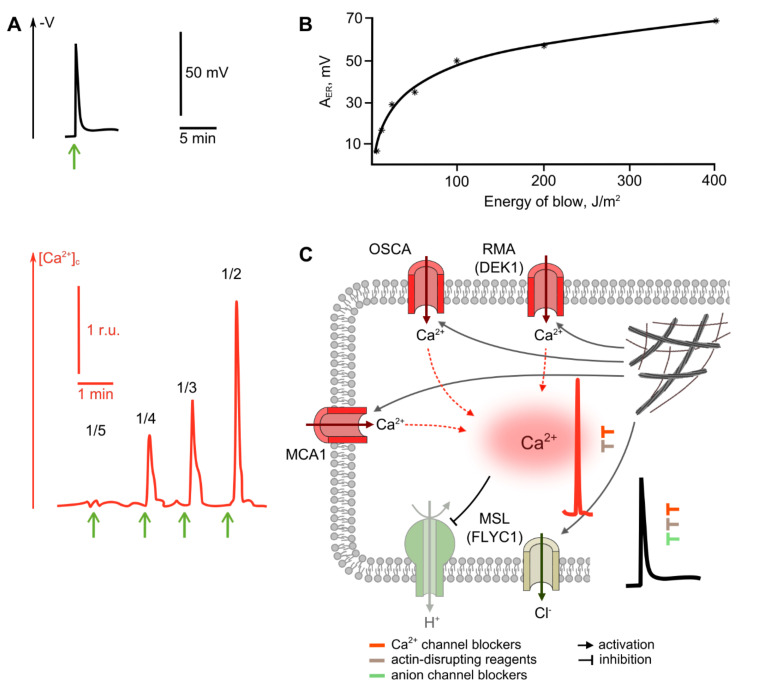
Electrical reaction (ER) induced by mechanical stimulus: (**A**) ER [25] and [Ca^2+^]_c_ dynamics [130] induced by mechanical stimulus (Copyright (1995) National Academy of Sciences, U.S.A.); (**B**) Dependence of the ER amplitude on the energy of blow [25]; (**C**) Hypothetical scheme of the generation of ER induced by mechanical stimulus. Explanations provided in the text. Black lines indicate the ER, red lines—[Ca^2+^]_c_ dynamics. V is the extracellular (surface) electric potential. Green arrows indicate the moment of mechanical stimulation. The numbers above the [Ca^2+^]_c_ peaks represent the wind force in N (r.u.).

Non-damaging mechanical stimulus triggers short-term transient depolarization, which has an impulse form [25,27,123,126,127] (Figure 4A). The amplitude of the ER depends on the strength of the mechanical stimulus, and when the threshold level is reached, the propagating AP is induced [125,128]. These features of mechanically induced ER allow referring it to the receptor potential. The noted features, namely the dependence of the response amplitude on the strength of the stimulus and the ability to induce AP, are also characteristic of the mechanically induced reactions of non-locomotive plants, both higher plants, and chara algae, the classical model object of plant electrophysiology. We should underline that the logarithmic dependence of the amplitude of mechano-induced ER on the strength of touching differs from the sigmoid dependence, with an exponential growth phase typical for cold-induced ER (Figure 4B). Of note is that stimulation equivalent to a falling rain drop is sufficient to generate an ER [25,129].

The formation of mechanically induced ER is mainly associated with the activation of ion channels, as evidenced by a significant decrease in the electrical resistance of the membrane [131]. Inhibitory analysis and experiments in varying the ionic composition of the medium indicate the role of calcium’s influx and the anions’ efflux at the depolarization stage [129,131,132,133]. Of importance, inhibition of calcium entry from the surrounding medium, both with the blocker of plasmalemma calcium channels, La^3+^, and the chelator EGTA, does not lead to the complete suppression of the ER [129,131,132,133,134], which is in line with the significant contribution of anions. Also, apoplast alkalization and cytosol acidification during a mechanically induced reaction have been reported, which are apparently caused by the transient inactivation of H^+^-ATPase [134,135]. The dynamics of pH change have been shown to well-match the dynamics of intracellular Ca^2+^; moreover, pH changes are suppressed by the addition of La^3+^ [135], which reveals the role of Ca^2+^ in modulating H^+^-ATPase activity. It should be noted that pH changes during the generation of a mechano-induced reaction are less pronounced than those for cold-induced ones [134], which, along with the significant drop in membrane electrical resistance mentioned above, indicates the decisive contribution of passive ion fluxes to its formation.

Calcium signatures, in the case of mechanical stimulation, are short-term spikes, the duration of which usually does not exceed a few seconds [36,130,135,136,137]. In some studies, the presence of a second, longer wave of Ca^2+^ was registered after a single mechanical stimulus, in particular, after root bending [135,138]. The amplitude of the calcium peak [130,136], similarly to the amplitude of changes in the electrical potential, depends on the strength of the mechanical stimulus (Figure 4A). LaCl_3_ inhibits the mechano-induced calcium spike, along with trap closure, when stimulating the sensory hairs of the Venus flytrap [139]. Nevertheless, it is likely that the main source of Ca^2+^ under mechanical stimulation is intracellular compartments, as evidenced by a more significant suppression of mechano-induced Ca^2+^ spikes by ruthenium red, an inhibitor of intracellular Ca^2+^ channels [130,136,140,141].

Along with the peak in intracellular Ca^2+^, a burst of ROS has also been also registered upon mechanical stimulation. In this case, the level of ROS production was less than under heating [78]. It was shown that the mechanically induced accumulation of ROS is attributed at least partially to RBOHC activity, has a dynamics similar to that of Ca^2+^, and is inhibited by La^3+^ [135].

Mechanosensitive ion channels, in particular, calcium channels, are considered primary receptors for mechanical stimuli. Thus, MCA1, from the family of proteins with Mid1-complementary activity, found in *Arabidopsis* root cells, is assumed to be responsible for the perception of soil hardness [142,143,144]. Also, the recently discovered rapid mechanically activated channel (RMA), encoded by the DEK1 gene and presumably located in the plasma membrane of epidermal cells is the probable mechanically sensitive Ca^2+^ channel involved. This channel is effectively inhibited by Gd^3+^, a known inhibitor of mechanosensitive channels in plants, and weakly inhibited by La^3+^ [145,146].

For *Droseraceae*, the involvement of DmOSCA (a homologue of OSCA/TMEM63 in *Arabidopsis*) in the induction of Ca^2+^ entry during mechanically induced ER generation has been suggested; however, this suggestion has not been experimentally investigated. Of note, along with sensory hairs, the increased expression of DmOSCA occurs in flowers and roots, which may indicate its general role in mechanoreception [124]. This assumption is supported by the data on the mechanoreception of model cells expressing the OSCA1.2 channel, its homologue in *Arabidopsis*. The operation of other channels of the OSCA/TMEM63 family in mechanical sensitivity, possibly with a higher activation threshold, also cannot be excluded [147].

Along with the channels mentioned above, Ca^2+^-channels regulated by phospholipase C are probably involved in the formation of a prolonged wave during root bending. The activation of this type of channel is associated with the functioning of the receptor-like kinase FERONIA (FER) and the peptide hormone RALF (rapid alkalinization factor), which is confirmed by the Ca^2+^-wave suppression by neomycin [148] and in FER-deficient *Arabidopsis* plants [138]. Mechanical stimulus can facilitate RALF–FER interactions by inducing RALF secretion or increasing the availability of FER binding surfaces for the peptide [138,149].

It is likely that not only Ca^2+^ channels can act as primary mechanosensors. Channels MSL9 and MSL10, from the MSL family, which exhibit predominantly anionic conductivity, are also considered as candidates for primary mechanosensors. These channels are expressed in roots [143,145,150,151]. The putative anion channel MSL8 can act as a mechanosensor in flowers during pollination [152].

In Venus flytrap (*Dionaea muscipula*) and other members of the *Droseraceae*, the msl flycatcher1 (FLYC1) gene was identified, which is most likely responsible for the organ-specific perception of a mechanical stimulus from prey in the sensory hairs of the trap. Its expression was many times higher in the sensory zone of hairs compared to other parts of plants; and structural modeling has demonstrated the ability of the FLYC1 protein to function as an anion channel. Tension-triggered electric currents have also been shown for model cells with an expressed FLYC1 gene [124].

The activation of mechanosensitive channels can be triggered either by the tension of the membrane, or by a change of the components of the cytoskeleton associated with it [51,153]. In the latter case, it is the cytoskeleton that is responsible for the primary perception of a mechanical stimulus, and perception is carried out by disintegration of its structure, which is perceived by mechanosensitive channels [51,140,141].

Experimental data indicate the necessity of a native actin cytoskeleton for Ca^2+^ entry [140,141,154]. Its destruction by latrunculin A leads to an increase in the intracellular concentration of Ca^2+^, similar to that induced by mechanical stimulation. The main Ca^2+^ store involved in the reaction are apparently intracellular compartments, as evidenced by the inhibition of Ca^2+^ entry by ruthenium red [140,141]. Along with actin filaments, microtubules also play an important role in mechanoreception by changing their orientation under mechanical stimulation [51,153].

To summarize, the sequence of events during the induction of mechanically induced ER can be represented as follows (Figure 4C). A mechanical stimulus leads to the activation of mechanosensitive channels by changing the structure of the cytoskeleton and/or changing the membrane tension. Stimulus-activated channels, the genetic nature of which are currently unknown—though it is assumed that they belong to the families MCA, OSCA, MSL—determine the influx of Ca^2+^ into the cell and the efflux of anions. The influx of Ca^2+^ leads to an increase in the intracellular concentration of Ca^2+^ and, together with the efflux of Cl^−^, to the generation of depolarization.

### 2.4. Salinity and Drought

The exposure of plants to soil salinity and drought is a worldwide problem that threatens crop production; thus, great attention is paid to studying how these stress stimuli affect plants. Recently, significant advances have been made in under-standing the mechanisms of the perception of salinity and drought by plants, including the induction and propagation of root-to-shoot and shoot-to-root signals, molecular mechanisms of adaptation and resistance. Several recent reviews have provided a comprehensive analysis of the state-of the art in this field [3,155,156], so, in our work, we will briefly summarize the most important points.

**Figure 5:**
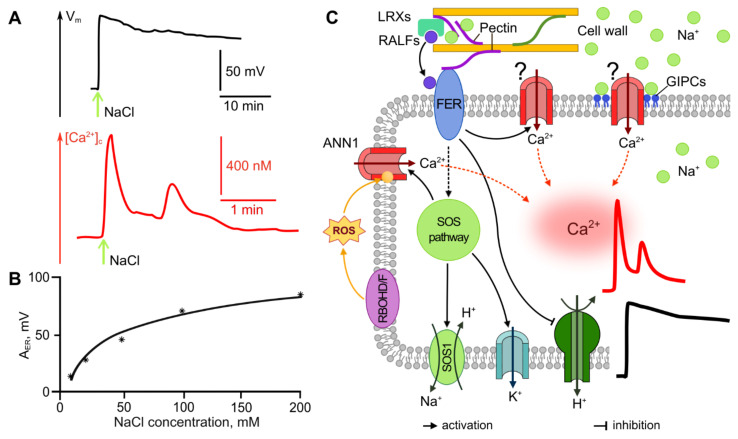
Electrical reaction (ER) induced by salt stress: (**A**) ER [162] and [Ca^2+^]_c_ dynamics [163] induced by salt stress; (**B**) the dependence of the ER amplitude on the NaCl concentration [157]; (**C**) a hypothetical scheme of the generation of ER induced by salt stress (based on [156]). Explanations are provided in the text. Black lines indicate the ER, red lines—[Ca^2+^]_c_ dynamics. V_m_ is the membrane potential. The green arrow indicates the moment of NaCl treatment.

The study of salinity- and drought-induced ER is commonly performed using treatment of plants with NaCl or osmoticum treatments. An over-optimal NaCl concentration in the solution bathing the roots causes membrane depolarization, the amplitude of which increases from several mV to about 100 mV with an increase in NaCl concentration in the range of 5–250 mM [157,158,159,160,161]. Not only ER amplitude, but also the velocity and start time of the depolarization depend on NaCl concentration [157,158]. The dependence of the amplitude of the salt-induced ER on the strength of the stimulus has a logarithmic character (Figure 5B), similar to mechanical stimulation (Figure 4B), and in contrast to the sigmoid dependence with an exponential growth phase that is characteristic of temperature (Figure 1C and Figure 2C) and light (Figure 3B) stimuli. The time interval between starting the stimulation and the ER occurrence decreases, while the velocity of depolarization increases, with rising NaCl concentrations [157,158]. The repolarization phase of the salt-induced ER is weakly manifested, the steady-state membrane potential exceeds the initial membrane potential by several tens of mV and is reached within tens of minutes (Figure 5A) [158,159,161,162]. It was reported that NaCl treatment can induce two-phase dynamics of membrane potential (at medium and high NaCl concentrations) with the first maximum being registered a few minutes after the stimulation and the second maximum after tens of minutes [157]. Under natural conditions, salt stress is not a short-term, but a long-term or constant stress factor, therefore, the results of long-term observations are of particular interest. It has been shown that root cells remain depolarized for several days under salt stress [158].

The information on drought-induced ER was entirely obtained from the experiments implying simulating the drought by osmoticum treatment [164]. In response to low water potentials, transient hyperpolarization of root cells with an amplitude of about 25 mV was observed for maize [165] and *Arabidopsis* plants [166]. The opposite effect was registered in response to high water potentials: depolarization with amplitude of about 40 mV [165,166].

As was shown above for other abiotic stimuli, calcium influx into the cell is one of the first events triggered by salt and drought stress. The dynamics of [Ca^2+^]_c_ represent a peak, the duration of which does not exceed several tens of seconds (Figure 5A) [163,167]. Of note, despite both drought and salinity leading to the same common negative effect—a decrease in water availability—there is a stimulus-specificity of the calcium signatures under these conditions [168]. In particular, the formation of a two-peak transient elevation of cytosolic Ca^2+^ in response to NaCl has been shown, in contrast to a one-peak response induced by an osmoticum treatment [163,167]. The amplitude of the calcium peak both under the NaCL and osmoticum treatments depends on the strength of the stimulus [163], similar to that for electrical potentials.

The mechanisms of salinity and drought perception are described, in detail, in other and comprehensive works [48,156,169]. Salt-stress sensing is associated with the functioning of several key molecular systems, including glycosyl inositol phosphorylceramide (GIPC) sphingolipids and LRXs-RALFs-FER complex [156,170,171]. GIPCs directly bind Na^+^ and regulate the Ca^2+^ influx into the cytosol via an unknown Ca^2+^ channel (Figure 5C) [171]. In the apoplast, cell wall-localized leucine-rich repeat extensins, LRXs, together with RALFs and FER, function as a system to sense salt stress-induced cell wall changes and trigger Ca^2+^-signaling (Figure 5C) [156,169,170]. GIPCs- and FER-mediated Ca^2+^-influx is required for the activation of the salt overly sensitive (SOS) signaling pathway [156].

In addition to the perception of salinity, FER is probably involved in drought-sensing, although in this case it is more likely to be involved in sensing the drought-induced damage to the cell wall [48,169]. The perception of drought and salinity is also associated with the activation of plasma membrane-localized Ca^2+^-channels of the OSCA1 family that respond to high extracellular osmotic potential or plasma membrane tension caused by water deficiency [48,172,173,174].

One of the earliest responses to the activation of salt- and drough-induced signaling pathways is the increase in [Ca^2+^]_c_ and the changes in the electrical potential described above. Increase in [Ca^2+^]_c_ is associated with the activation of unknown Ca^2+^-channels regulated by GIPC and FER, as well as with the functioning of OSCA1 and ANN1 channels, the activation of which is controlled by RbohD/F [79]. The RALF–FER-induced inactivation of H^+^-ATPase, along with the SOS pathway-regulated activity of the Na^+^/H^+^ antiporter SOS1 and the K^+^-channel AKT1 of the plasma membrane, probably contribute to the change in the electrical potential induced by the salinity [156].

Along with changes in the electrical potential observed in the stimulation zone, water and salt stress induce propagating root-to-shoot signals, including hydraulic signals, ROS and Ca^2+^ waves, and electrical signals [3,155]. In particular, drought induces the VP propagation to the shoot of *Vitis vinifera* [175]; and a wave of depolarization that propagates along the phloem in *Zea mays* [176]; irrigation after drought induces the AP propagation in the phloem [176,177].

Salinity and drought cause changes in electric potentials in leaves, which can be considered a consequence of the propagation of signals of different nature, including hydraulic and ROS–Ca^2+^ waves [3,155]. It should also be noted that, in the case of salt stress, Na^+^ is loaded into the xylem and transported to the leaves [156,178]. The possible direct role of Na^+^ as an inducer of electrical responses in the shoot is confirmed by the fact that moderate concentrations of NaCl cause depolarization of mesophyll cells comparable in amplitude the depolarization of root cells [179,180].

Water stress in roots also induces an electrical response in leaves. Drought leads to depolarization of leaf cells in various plant species [176,181,182,183]. The change in electrical potential can gradually develop as the soil-water content decreases [176]. Soil rehydration also induces an electrical response in the form of the hyperpolarization of leaf cells [176,181,182]. For example, in maize leaves, hyperpolarization with an amplitude of about 50 mV was registered 10 min after soil watering [176]. In an inverted reaction to watering, a depolarization with an amplitude of about 100 mV was obtained for *Vitis vinifera* at a considerable distance (150 cm) from the stimulation area at the roots [10]. In general, the available data convincingly indicate the presence in plants, including woody ones, of root-to-shoot communication based on the transmission of rapid long-distance signals.

Thus, the electrical reactions of plants induced by salinity and drought are associated with the functioning of a number of molecular systems. Primary salt-stress sensors, represented by GIPC and the LRXs–RALFs–FER complex, activate the signaling pathway that includes calcium influx and the regulation of the activity of H^+^-ATPase and K^+^-channels, which probably results in a change in electrical potential. Salinity and drought, along with the induction of changes in the electrical potential in the stimulated area, cause the propagation of long-distance root-to-shoot signals, represented by hydraulic, electrical and ROS-Ca^2+^ waves.

### 2.5. Wounding

There is quite extensive information about the presence of electrical reactions induced in plants by local wounding. Under natural conditions, local wounding can be caused by an attack by herbivores, caterpillars, etc. Without considering the biotic stressors, we focus, in this review, on mechanical damage and burning, which are used as typical VP inducers in a wide number of studies [17,184,185,186]. Obviously, measuring the potential directly in the destroyed cells in the damaging area is impossible; so, in all studies, ERs propagating from the wounded area are registered. The wounded area is a source of signals that propagate to the intact tissues and organs of the plant. These signals include, first of all, a hydraulic wave and chemical signals [6,187]. A hydraulic wave, in the case of wounding, arises due to a combination of at least two factors: the release of vascular juices from damaged cells into the xylem vessels, and an increase in ionic concentrations [188,189,190]. A damaging agent, such as a burn, additionally causes an increase in gas volume and pressure in the intercellular spaces and in the xylem [16,191]. Together, this leads to the propagation of a hydraulic signal, which manifests itself in a rapid increase in xylem pressure [16].

The chemical signal represents the release of damage-related molecular patterns (DAMP). Primary DAMPs include ATP, fragments of the cell wall or enzymes that destroy the cell wall, and other compounds that normally play their physiological role in intracellular homeostasis and are found outside the cell only when it is damaged, causing the induction of a defense response. Secondary or inducible DAMPs are endogenous molecules that are actively produced or modified during cell death and act exclusively as signals [187,192,193]. Both under mechanical damage and burn, multiple DAMPs are released from damaged cells; we will focus on those for which the possibility of inducing ER has been shown with a high degree of probability.

Glutamate (GLU) is considered to be one of the likely damage-associated inducers of ER, in light of the fact that its receptors, GLR3.3 and GLR3.6, have been shown to participate as Ca^2+^ channels contributing to the formation of distant ES [18,194,195].

Oligogalacturonides (OG) are released from the cell walls as a result of the homogalacturonan fragmentation by polygalacturonases (PG). OGs are recognized by wall-associated kinase 1 (WAK1) and WAK2 receptors, transmembrane proteins that bind also polygalacturonic acid and pectins [187,192,193,196], which recognition causes a rapid (i.e., within a few minutes) increase in [Ca^2+^]_c_ and the Ca^2+^-dependent activation of NADPH oxidase, leading to a burst of H_2_O_2_ in the vessels of the plant [197].

ATP, released upon cell damage, is recognized by a family of P2 receptor kinases 1 (P2K1) located on the plasma membrane, most probably a kinase similar to the L-type lectin receptor I.9 (LecRK-I.9) [193,198,199], leading to a Ca^2+^-mediated increase in NADPH oxidase activity (RBOHD) by phosphorylation and a burst of ROS [198,200].

NAD^+^ and NADP^+^ are also capable of acting as primary DAMPs; lectin receptor kinases LecRK-VI.2 and, possibly, LecRK-I.8 (for NAD^+^) can be their receptors [187,192,193,201]. The formation of a complex of activated receptor with the brassinosteroid insensitive1-associative1 kinase1 (BAK1) coreceptor triggers a defense response, however, many aspects of its formation are not yet clear [193,202]. Nevertheless, it has been shown that this response induction requires the participation of Ca^2+^ [203].

A number of proteins, such as systemin and protein elicitor peptide 1 and 3 (Pep1 and Pep3), act as secondary DAMPs, whose production is activated in response to the release of intracellular precursors or synthesis activators. The receptors have been identified for systemin and Pep1: systemin receptor 1 and 2 (SYR1 and SYR2), and receptor-like kinases PEP receptor 1 and 2 (PEPR1 and PEPR2), respectively. It should be noted that the production of secondary DAMPs is mostly observed tens of minutes after damage [187,192,193], which does not imply their contribution to the induction of fast electrical reactions. However, Ca^2+^-dependent production of Pep1 was registered less than one minute after damage [204]. It is also reported that Pep1 application causes activation of Ca^2+^-channels [205]. Pep1-dependent activation was shown for CNGC2 [206] and CNGC19 [207].

It can be assumed that the sequence of events in response to wounding is as follows: cell damage causes the release of a variety of primary and secondary DAMPs, including glutamate, OG, Pep1, and ROS, which can lead to the activation of ligand-sensitive Ca^2+^ channels. Simultaneously, a hydraulic wave is induced upon damage, which can activate mechanosensitive Ca^2+^ channels. The influx of Ca^2+^ leads to the induction of membrane depolarization in the intact cells. Thus, the spreading of DAMPs and the propagation of hydraulic waves can serve as an inducer of ES in intact cells.

In general, it can be stated that plants have specialized receptor systems, the activation of which by external stimuli leads to the formation of a local ER in the stimulation area. The key role in the generation of a local ER is apparently played by calcium’s entry into the cell, the signatures of which correspond well with the dynamics of the electrical potential. Local ERs induced by various stimuli have a number of common features, which include the dependence of the ER amplitude on the strength of the stimulus, desensitization upon repeated stimuli, and the ability to induce propagating ES. However, local ERs exhibit specific features under the action of specific stimuli. 

They include the parameters of the dynamics of electrical potential and [Ca^2+^], which originate from the peculiar mechanisms of the perception of stimuli. In addition, stimulation can induce, along with electrical, other types of long-distance signals, including hydraulic and chemical signals, which are capable of cross-interacting with electrical potentials along the path of their propagation.

## 3. Parameters of Distant Electrical Signals Induced by Different Stimuli

Considering the possibility of transmitting information in plants with the participation of distant electrical signals, it is necessary to note the presence of three types of them, namely, APs, VPs and SPs [1,6,8,11]. The last are not covered in this review due to the small number of experimental studies concerned with them. Basic information about all types of ES is summarized in Box 1. The presence of different types of ES in plants increases their ability to transmit information by means of triggering the corresponding type of signal by a certain type of stimulus. Indeed, when viewed in general, there is specificity in the propagation of a given type of electrical signal relative to its inducing stimuli (Table 1). Thus, AP generation is observed in response to a variety of mostly non-damaging influences, such as cold, electric current, changes in illumination, and mechanical touch (Table 1). On the contrary, VP generation occurs under the action of damaging stimuli [1,6,10], such as mechanical damage, burning, and heating to high temperatures (Table 1). However, detailed analysis reveals a more complex picture. For example, in case of mechanical damage, whether as slight as a needle prick or much more serious, such as cutting off a part of a leaf, an AP-like signal is always generated [28,208,209]. Also, the signal in the *Dionea* trapping leaf [58] is classified as an AP, as well as the signals induced in beans (*Vicia*) [53] and potatoes (*Solanum*) [210] by a temperature rise, which is commonly considered a typical VP-inducer.

Along with “simple” reactions, represented by a fast AP impulse or a slow wave of the VP potential, the possibility of the propagation of complex combined reactions must be mentioned. The first type of such complex reactions is a slow potential wave typical of VP, against the background of which fast electrical pulses arise [195,223,235] denoted in some works as an “accompanying” AP. Another type of complex reaction represents, apparently, independently propagating APs and VPs, which can be observed, for example, in a leaf that has been burned [232,239] or mechanically damaged [228]. In this case, a clear separation of the complex reaction into two components takes place with an increase in the distance from the stimulation zone, due to different speeds of signal propagation—APs propagates ahead of VPs [235,239].

In general, it can be assumed that there is a given specificity for the type of propagating signal, depending on the nature and intensity of the abiotic stimulus.

**Figure 6:**
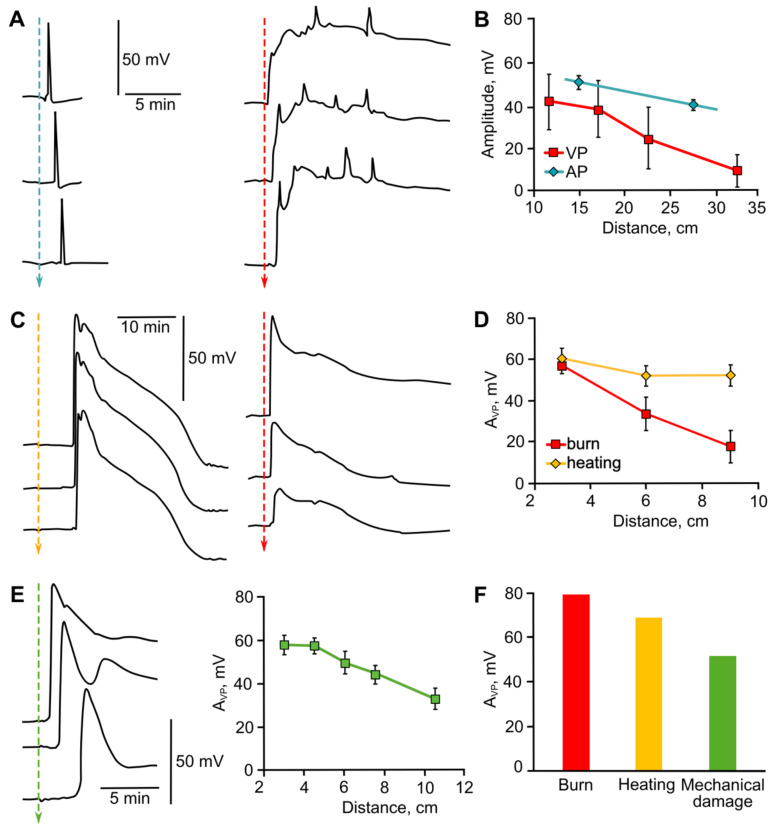
Long-distance electrical signals induced by different stimuli: (**A**) an electric current-induced action potential (AP) and a burn-induced variation potential (VP) in a sunflower stem [218]; (**B**) the dependence of AP and VP amplitudes on the distance from the stimulated area [218]; (**C**) burn- and heating-induced VPs in a wheat leaf [54]; (**D**) the dependence of the amplitudes of VPs caused by burn and heating on the distance from the stimulus [54]; (**E**) a VP, induced by mechanical damage in a pea stem, and the dependence of its amplitude on the distance from the stimulated area [226]; (**F**) the amplitudes of VPs induced by burning, heating and mechanical damage. Averaging was performed based on the data from the works in Table 1. Dashed arrows indicate the moment of stimulation by electric current (blue)/burn (red)/heating (yellow) and the electrical signal propagation direction. Data are represented as typical records (**A**) and (**C**) and mean ± SEM (**B**) and (**D**).

Another aspect that needs to be considered is the specificity of the parameters of ES of the same type but induced by different stimuli. Despite the variety of stimuli that induce APs, we can state the relative constancy of AP parameters, regardless of the type of inducing stimulus [27,28,215,216,218]. As an example, Figure 6A,B demonstrates AP amplitudes and their dependencies on distance, which are almost identical for electrical stimulation and cooling, as well as the absence of a decrease in AP amplitudes during propagation [218]. The absence of stimulus-specific traits is due to the nature of APs. An AP in plants is a self-propagating electrical signal that obeys the “all-or-nothing” law, similar to APs in animals. That is, an excitable cell in which the threshold level of depolarization is reached will generate a standard electrical pulse, and its amplitude and duration will not depend on the parameters of the stimulus [6,253,254].

Though, the species-specific features of APs should be noted, which are expressed mainly by differences in the duration and speed of pulse propagation for locomotor and non-locomotor plants [8].

The duration of APs in locomotive plants is only several seconds, at a propagation speed of up to 8–9 cm/s, while in non-locomotive plants the duration reaches tens of seconds at a propagation speed of less than 3 cm/s [6,8,11,254]. There are also a few works showing a certain variability of AP parameters, depending on the direction of propagation and the organ from which the parameters are recorded [28,214].

In general, it can be presumed that an AP spreading within one organ or tissue outside the area of local stimulation does not have stimulus-specific features in a plant of a given species. This electrical signal carries information about the fact of the stimulation, but not about its nature, beyond its belonging to AP-, and not VP-inducing stimuli.

An absolutely different picture is observed in the case of VPs. First of all, it is necessary to note the significant variability of the parameters of VPs induced by various stimuli—the reason why the variation potential has its name. Mechanical damage, such as cutting off the root [223,225], crushing the leaf [226] or dissection [18,227] often causes VPs in the form of an extended pulses with durations of several minutes The reaction to burning, with a duration of up to tens of minutes, has an irregular form, and its extremely slow repolarization often does not lead to a full recovery of potential during a registration time that usually exceeds half an hour [54,185,186,235,238,239,243,245,246] (Figure 6E). In the case of gradual heating, the reaction is also characterized by a long duration, but of a more regular form in comparison with burning [54,230] (Figure 6C). A comparison of the parameters of VPs induced by stimuli of different natures reveals that the VP amplitude in the immediate vicinity of the wounding area is maximal for burning, and somewhat less for heating and mechanical damage (Figure 6F). Moreover, the dependence of the VP’s amplitude on the area of the wound [222,235] and the intensity of the stimulus [255] has been reported.

Along with the analysis of the VP parameters themselves, such as the amplitude and duration, it is also important to consider their changes as the reaction propagates. The decrement in the amplitude and speed of a VP propagation is commonly considered a feature that distinguishes it from an AP [6,9,254]. Of note, the existence of a decrement is in good agreement with the concept that a VP is not a self-propagating electrical signal, but is a local electrical reaction induced by a hydraulic or chemical signal [6,9,254] (Box 1). A comparative analysis of the decrement of VPs induced by different stimuli (Figure 6B,D,E) demonstrated the presence of a pronounced ES attenuation after burning and mechanical damage, whereas, under heating, the amplitudes of VPs in fact did not decrease [54,218,226,235]. Thus, it can be noted that VPs exhibit a certain stimulus-specificity.

What determines the stimulus-specificity of VP parameters? The mechanisms of the specificity of VPs in plants have to be elucidated in future studies, but they can be hypothesized, based on the mechanisms of propagation of this distant stress signal. VP propagation is quite complex, due to VPs being composite signals of cross-interacting electric, hydraulic and chemical waves [3,6,17,175]. A hydraulic wave can act as a trigger for electrical changes during VP propagation, which is convincingly evidenced by VP induction using an artificial increase in pressure [190,255]. Hydraulic wave propagation induces VP generation through the activation of mechanosensitive Ca^2+^-channels [6,16,17], assumedly from the OSCA1, MSL, or MCA families, although there is no definitive data on the genes involved in the reaction at present [14,256].

The chemical mechanism of VP propagation is based on the spread of the “wound substance”, the so-called Rick’s factor, through the vessels from the wounding area. In this regard, the “ROS-Ca^2+^-hub” concept must be noted, which implies the transmission of self-sustained ROS and Ca^2+^ waves. According to the concept, Ca^2+^-activated NADPH oxidase RBOH interacts with ROS-activated Ca^2+^-channels to generate and enhance stress-induced Ca^2+^ and ROS signals [2,3,14,256]. RBOH is a membrane-bound enzyme that uses NADPH as an electron donor and produces a superoxide in the apoplast, which is rapidly dismutated to hydrogen peroxide (H_2_O_2_). An increase in cytosolic [Ca^2+^] activates RBOH and enhances H_2_O_2_ production, while H_2_O_2_, in turn, activates Ca^2+^ influx via ROS-activated channels. Thus, this mechanism provides self-maintenance of the propagating ROS-assisted Ca^2+^ wave [1,8,14,198,200,257,258]. The propagating wave of hydrogen peroxide (H_2_O_2_) can be considered the above-mentioned “wound substance” causing the VP’s generation. The systemic propagation of H_2_O_2_ has been shown for mechanical damage, heat and light shocks [78,118,259]. Peroxide-triggered VP generation may be attributed to the activation of GLR family calcium channels [18,19,194,195]. This hypothesis is supported by the propagation of the wound-induced Ca^2+^ wave along the vessels of the plant, with parameters correlating to that of the electrical signal [19,194]. It should be noted that velocity of transmission of chemical signal-inducing VPs can be significantly increased by the propagation of a hydraulic wave [17]. 

The most likely explanation of the stimulus-dependent changes in VP parameters owes to the different contributions of the individual components to the complex signal. For example, under heating, chemical signals are likely to dominate. Large amounts of ROS released in the heated area, apparently, results in the formation of a self-sustaining ROS wave, providing non-decremental VP propagation (Figure 6). In the case of mechanical damage and burning, a hydraulic wave could prevail. This is indicated by the higher initial velocity of the VP induced by these stimuli in comparison with heating [226]. On the other hand, there is a relatively small wound area with these modes of stimulation, which does not provide the release of a sufficient amount of wound substance spreading along the vascular bundles. The inability of a relatively small amount of ROS to induce self-sustaining propagating wave leads to attenuation of VP with distance (Figure 6).

When considering propagation of all types of ES, it is necessary to note the shifts in the concentrations of calcium and protons in cell cytoplasm and apoplasts associated with their generation [6,8,254]. It is known that the calcium signature determines the peculiar features of the functional response induced by the distant signal, in particular, gene expression and hormone production [8,37,42]. No data has been reported on the role of the pH signature developed due to the transient suppression of H^+^-ATPase activity. However, good agreement was shown between the dynamics of pH during VP generation and the dynamics of the VP-induced photosynthetic response [4,11,54,184]. In turn, the signatures of calcium and pH are presumably similar to the dynamics of the electric potential. Therefore, despite the fact that the features of the Ca^2+^ and pH signatures during the ES propagation have not yet been studied, it can be assumed that they have features similar to those for ES [1,6,8,9,10,11,17].

In general, we can state the presence of characteristic features in distant ES in plants determined by the nature of the stimulus. First of all, a certain type of distant ES, represented by APs, VPs and SPs, spreads in response to the action of certain (“own”) stimuli. Therefore, the propagation of a given ES informs the non-stimulated parts of the plant not only about the fact of stimulation, but also about the assignment of the stimulus to a certain category. The presence of the stimulus-specific features of VPs can be potentially responsible for the transmission of additional information about the nature of the stimulus. This ability to possess specificity originates from the complex nature of VPs, which is a combination of interacting electrical, hydraulic and chemical signals. The cross-talk of various signals is considered, today, to be the primary means of transmitting information in plants [3,6].

## 4. Specificity of Systemic Responses Induced by Electrical Signals under the Actions of Different Stimuli

Despite the long history of studying ES in plants, it is still not entirely clear whether plants are capable of decoding signals and specifically responding to the stimuli that caused them, or whether they are more likely to develop a universal stress response that leads to the formation of nonspecific systemically acquired acclimation. The best-studied ES-induced responses are those observed with the naked eye in specialized structures and organs, where such signals have a clearly defined function and specific target cells/tissues. Such specialized structures, first of all, include the motor organs of insectivorous and sensitive plants: the folding leaves of mimosa, the traps of the Venus flytrap, sundew, etc., where APs, induced in receptor areas, trigger the rapid folding of the leaves or trap closure, followed by the production of digestive enzymes [27,123,125,127,132,208,219].

AP generation in the reproductive organs of plants can be considered another example of “narrow specialization”. It is known that APs occur in flowers’ pistils a few minutes [26,260] or several hours [261] after the ingress of pollen of the same species at the stigma. The AP resulting from pollination triggers a number of processes, including the activation of respiration and the accumulation of ATP and starch, which increases the energy supply of the ovary and the likelihood of fertilization [26,260]. The effect of APs on other fertilization processes has also been reported. For example, in mimulus and incarvileia, APs promote the formation of a moist chamber from the stigma blades, in linden, APs stimulate nectar formation and secretion, and in fern gametophytes, APs induce the guttation necessary for fertilization [15].

Apparently, an AP itself does not carry information about the stimulus that caused it. This is confirmed by the similarity of responses induced by APs that are, themselves induced by different stimuli. Trap closure, in the Venus flytrap, occurs due to the propagation of APs induced not only by the contact by trigger hairs with the prey, but also by mechanical and electrical stimuli and changes in illumination and temperature [33,99,100,262]. In mimosa, leaf folding occurs not only following the touch-induced AP, but also following an AP induced upon wounding, cooling, heating, changes in illumination, electrical stimulation, or ionizing radiation [140,263,264]. The absence of a strict relationship between the type of stimulus causing AP generation and the AP-induced response in the non-stimulated parts of the plant is also shown at the level of gene expression. For example, APs caused by electrical stimulation, mechanical damage or heating lead to an increase in the expression of the proteinase inhibitor 2 (pin2) gene with similar dynamics [210,215].

Despite the identical parameters of APs obeying the “all-or-nothing” law, plants have acquired the ability to develop stimulus-specific functional responses using this type of signal. First, the probability of the AP’s occurrence and propagation may vary under the action of different stimuli. Thus, during fertilization, the probability of AP propagation is higher in the case of cross pollination than in case of self-pollination, which contributes to the genetic diversity of the offspring. The pollen of plants of other species is not able to induce an AP [261]. Mechanical stimulation of the stigma without pollen triggers an AP, but it does not reach the ovary; thus the process of preparing for fertilization does not start [260].

Similarly to animals, plants can also use frequency coding to transmit information. Plants have the ability to decode information based on frequency modulation and the number of propagating APs. When an insect visits the *Dionaea muscipula* trap and stimulates its mechanoreceptors, APs are generated. The trap closes if at least two APs were induced by touching trigger hairs within a maximum of 30 s. Additional APs cause a stronger and more prolonged closure of the trap and trigger the synthesis of jasmonic acid (JA). When the number of APs reaches five, the expression of genes coding hydrolases is triggered in order to digest the prey [219,265]. Another insectivorous plant, the sundew, is capable of generating APs both upon the capture of prey and upon mechanical stimulation, but feeding causes a series of APs, whereas a mechanical stimulus causes a single AP. As a result, in both cases, the trap-bending reaction occurs, but a different amount and distribution of jasmonates and digestive enzymes over the trap is observed [208].

Apparently, the spatial coding of information transmitted by means of AP can take place along with frequency coding. For example, the trap-bending reaction and the accumulation of jasmonate in the sundew occur in different ways, depending on the localization of the stimulated tentacles (central or marginal). If the prey falls into the middle of the trap, the marginal tentacles bend towards the center. This is assumed to be governed by the following sequence of events: the induction of a series of APs near the central tentacles, followed by the gradual potential and the accumulation of jasmonates, and then the movement of the marginal tentacles. If the prey falls on the edge of the trap, the marginal tentacles quickly bend one by one, and this movement is induced by their individual series of APs of a certain pulse number and frequency [208].

The frequency and spatial decoding of signals transmitted by means of an AP is, probably, inherent only in highly specialized (mainly locomotor) organs. In a more common situation, a stimulus causes the propagation of a single AP in non-specialized organs, which induces a universal stress response associated mainly with a temporary retardation in plant growth and development, a deceleration in metabolism, and the synthesis of defense-related compounds. Of note, even in specialized organs of insectivores e.g., in the traps of the Venus flytrap and sundew, the AP propagation induced by touching the trigger hairs, along with movement, causes a decrease in photosynthetic activity [208,265,266].

To fine-tune the strategy of adaptation to certain stressors, plants can potentially use different types of ES, namely APs and VPs. A comparison of responses to AP and VP is described below. First of all, it should be emphasized that both types of signals, regardless of the nature of the inducing stimulus, can trigger functional responses associated with defense and adaptation, which include:

- the temporary inhibition of photosynthesis (AP: [26,208,266,267]; VP: [11,213,214,238])
- increased respiration (AP: [268]; VP: [53,246,269])
- the accumulation of ATP (AP: [26]; VP: [246])
- the increased expression of pin2 gene (AP: [210,215]; VP: [215])
- the launching of downstream signaling cascades, primarily a change in the amounts of hormones (AP: [208,270]; VP: [18,234,236,243]).

Usually, functional responses induced by different types of ES demonstrate qualitative similarity, but, at the same time, there are quantitative differences in the amplitude and dynamics of the induced changes. Thus, a significant accumulation of callose in the sieve tubes (during apoplastic loading) was observed after the propagation of burn-induced VPs, while after a cutoff-induced AP, callose was deposited in smaller amounts and at a shorter distance from the stimulated zone [209]. Significant differences were observed in the production of metabolites, such as starch, sucrose, malate and others for maize, for VPs induced by cutting off leaf tips and for cold-induced APs. In both cases, the suppression of phloem transport was observed, which was more pronounced after cold-induced APs [213]. In tomato plants, burn-induced VPs cause approximately 1.5 times greater accumulation of chloroplast mRNA binding protein (CMBP) compared with cutoff-induced APs. At the same time a slight difference in the temporal characteristics of CMBP accumulation was observed [186].

Along with quantitative differences, some studies report qualitative differences between AP- and VP-induced responses. Thus, VPs induced by cutting off the tip of a maize leaf caused a decrease in CO_2_ assimilation and a decrease in transpiration, while no response was recorded during cold-induced APs [213]. In poplar, burn-induced VPs exclusively caused the suppression of the activity of photosystem II, and a decrease in CO_2_ assimilation, while a cold-induced electrical signal of the AP type did not induce these changes [214].

Along with the specificity of responses induced by different types of distant ESs, a given specificity is observed for a single separate signal in the form of a VP due to the above-mentioned dependence of its parameters on the type and intensity of the stimulus. Stimulus-specific response features are described, for example, for hormone shifts. Thus, it was shown that the local action of high light (HL) on *Arabidopsis* lead to a systemic increase in the concentrations of JA and salicylic acid (SA), while local heat stress (HS) caused a change in the content of SA only. The dynamics of the SA content are similar for both stimuli [5]. Multidirectional responses of stomatal guard cells are also reported for HL and HS: HL causes stomatal closure, while HS leads to stomatal opening [5]. The dissimilarity in responses can be resulted from differences in the dynamics of JA, which is known to induce stomatal closure [271].

It has also been shown that mechanical damage and HL lead to an increase in abscisic acid (ABA) response transcripts and JA response transcripts, while HS increases ABA response transcripts only in *Arabidopsis* [272]. The authors also demonstrated differences in changes in the content of metabolites (sucrose, amino acids, etc.) induced by the studied stimuli [272].

It can be assumed that the aforementioned specificity of VP-induced physiological responses is results from the differences in VP parameters induced by different stimuli. In a number of works, the relationship between the VP parameters and the parameters of the following VP-induced response has been shown. For example the amplitudes of the photosynthesis and transpiration responses correlate with the amplitudes of the inducing VPs [11,54,242]. A correlation between hormonal responses and VP parameters was also registered [18,236,243]. Such correlation is also observed in the case of the propagation of VPs with signal attenuation. The decrement in the burn-induced VP amplitude and a concurrent decrement in the VP-induced photosynthetic response have been observed with an increase in the distance from the stimulation zone [54].

To summarize, there is a plenty of evidence that a functional systemic response can depend on the parameters of the ES causing it. How can such dependence be mediated? It is established that the ES-triggered induction of a functional response is associated, first of all, with shifts in the concentrations of Ca^2+^ and H^+^ [1,6,8,11]. It can be assumed that the specific signatures of Ca^2+^ and H^+^ are responsible for the development of specific responses. The dependence of the response parameters, namely gene expression, on the parameters of calcium signatures has been shown; also, a model that correctly predicts this response, based on calcium signatures, has been developed [273]. To date, the extent of differences in the Ca^2+^ and H^+^ signatures associated with the propagation of VPs caused by different stimuli has not been studied. Nevertheless, a reliable similarity of the dynamics of pH changes and the dynamics of the electric potential during the generation of VPs [17,54,245] should be noted. This can probably be attributed to the dynamics of intracellular Ca^2+^ [269]. Since changes in the electric potential, i.e., VP shape, amplitude and duration, depend on the type of stimulus inducing it, stimulus-specific signatures of Ca^2+^ and H^+^ are strongly expected to be observed in cells in which VP generation has occurred. The specificity of the VP-induced responses may also be due to the complex nature of this long-distance signal, which includes not only electrical but hydraulic and chemical components [6,17]. Therefore, along with the pH and Ca^2+^ signatures, additional chemical compounds can contribute to the specificity of the generated response, which can be mediated by their perception by specific receptors and the subsequent activation of signaling pathways. The role of recognition of specific elicitors by plants has been well studied for biotic stimuli, e.g., during the formation of a pathogen-specific or insect-specific defense response [205,227]. However, such stimulus-specific DAMPs are also assumed to be involved in the induction of responses to abiotic stimuli, for example, in response to drought [173]. In general, the integration and interaction of hydraulic, chemical, and electrical signals provides great opportunity for the induction of stimulus-specific functional responses in plants. One of the possible ways of generating such a specific response is provided by the selective activation of Ca^2+^-channels by each of the components (or the ratio thereof) of the complex long-distance signal. Thus, a hydraulic wave can be independently perceived by mechanosensitive channels, including those on chloroplasts, directly regulating the photosynthetic response [3].

Thus, plants use several ways of inducting a specific systemic response, either independently or complementarily. The first is based on frequency coding and decoding of information transmitted by means of an AP. The second is associated with the existence of several types of long-distance ESs, which are characteristic of a certain category of stimulus. Finally, the transmission of information by means of a single signal, in the case of VPs, can be based on the specificity of its parameters, which depend on the nature and strength of the stimulus.

## 5. Conclusions

It is obvious that plants are able to adequately respond to changes in the environment. To date, electrophysiological studies of the reception of various abiotic stimuli have been performed; however, the molecular mechanisms underlying such reactions remain considerably unexplored. It also relevant to study not only the perception of each individual stimulus, but also a combination of stimuli which act simultaneously or sequentially, as is more typical for natural habitats. The ability of plants to transmit several types of distant signals makes it possible to encode information about the nature and/or intensity of the stimulus. Such general coding, which represents the categorization of stimuli, can be supplemented with finer and more informative coding due to the modulation of the ES frequency, in the case of APs, or the parameters of a single signal, in the case of VPs. In order for progress to emerge in the study of the specificity of long-distance ES, the genetic identification of ion transporters, primarily, ion channels, as the main contributors to ES formation, and the study of their mechanisms of function, is crucial. This will also contribute to understanding the possible mechanisms of the decoding of information transmitted by long-distance ESs, since shifts in Ca^2+^ and H^+^ concentrations play an important role in the induction of a systemic response. The specific signatures of calcium and pH, presumably, may underlie a given systemic response. Also, the formation of a stimulus-specific systemic response may be orchestrated by the cross-talk of distant stress signals of various nature, including electrical, hydraulic and chemical signals; however, such mechanisms have not been deciphered yet. Resolving these questions will potentially expand our knowledge on “decision-making” in plants in response to changes in the environment.

## Figures and Tables

**Table 1:** Overview on electrical signal types and the underlying stimuli from literature.

Stimulus	Plant	Signal	Reference(s)
Cold	*Mimosa*	AP	[27,211]
	*Zea*	AP	[212,213]
	*Biofhytum*	AP	[28]
	*Hibiscus*	AP	[26]
	*Arabidopsis*	AP	[18]
	*Populus*	AP	[214]
Electrical	*Lycopersicum*	AP	[215,216]
	*Luffa*	AP	[217]
	*Aldrovanda*	AP	[123]
	*Helianthus*	AP	[218]
	*Drosera*	AP	[127]
	*Biofhytum*	AP	[28]
	*Zea*	AP	[212]
	*Mimosa*	AP	[27]
Mechanical touch	*Dionaea*	AP	[132,219,220]
	*Aldrovanda*	AP	[123]
	*Drosera*	AP	[127,208,221]
	*Mimosa*	AP	[27]
Mechanical wounding	*Biofhytum*	AP	[28]
	*Vicia*	AP	[209]
	*Lycopersicon*	AP	[186,222]
	*Drosera*	AP + VP	[208]
	*Cucumis*	AP + VP	[223]
		VP	[55]
	*Arabidopsis*	AP + VP	[195,224]
		VP	[18]
	*Pisum*	VP	[223,225,226]
	*Phaseolus*	VP	[227]
	*Cucurbita*	VP	[228]
	*Mimosa*	VP	[229]
	*Zea*	VP	[213]
Heating	*Solanum*	AP	[210]
	*Vicia*	AP	[53]
	*Bidens*	VP	[230]
	*Cucumis*	VP	[55]
	*Pisum*	VP	[226]
	*Triticum*	VP	[54]
Burning	*Hordeum*	AP + VP	[231,232]
	*Lycopersicon*	AP + VP	[233,234,235]
		VP	[186,216,222,236]
	*Mimosa*	AP + VP	[235]
		AP	[27]
		VP	[185,237,238]
	*Vicia*	AP + VP	[209,232,235,239]
		VP	[209]
	*Cucurbita*	VP	[240]
	*Glycine*	VP	[241]
	*Helianthus*	VP	[190,218]
	*Populus*	VP	[214]
	*Pelargonium*	VP	[242]
	*Pisum*	VP	[226,243,244,245,246,247]
	*Nicotiana*	VP	[248]
	*Triticum*	VP	[54,189,249,250]
	*Zea*	VP	[251]
Pollination	*Hibiscus*	AP	[26]
Light	*Dionaea*	AP	[100]
	*Mimosa*	AP	[185]
Re-irrigation	*Persea*	AP	[252]
	*Vitis*	AP	[175]
	*Zea*	AP	[176,177]

Action potential (AP); variation potential (VP).
